# Bioaccessibility of Lead and Arsenic in Mining Waste and Mining-Affected Soils

**DOI:** 10.3390/toxics14020114

**Published:** 2026-01-26

**Authors:** Valérie Cappuyns, Lisa Dries

**Affiliations:** KU Leuven, Centre for Economics and Corporate Sustainability, Warmoesberg 26, 1000 Brussels, Belgium

**Keywords:** arsenic, bioaccessibility, lead, mineralogy, mining

## Abstract

In vitro bioaccessibility tests are used to estimate the release of contaminants from environmental samples during simulated digestion, making them available for intestinal absorption. In most cases, the samples are fine-grained materials with varying chemical, physical, and mineralogical properties, but it is not always clear how these properties influence the bioaccessibility of elements. The present study focusses on the bioaccessibility of lead (Pb) and arsenic (As) in mining waste and mining-affected soils. From the literature, data from mining waste and mining-affected soil samples were used to investigate the relation between chemical (element composition, pH, organic carbon content), physical (grain size distribution), and mineralogical properties of the samples and the gastric and intestinal bioaccessibility of Pb and As. Mean gastric As bioaccessibility was significantly lower in acidic samples than neutral and alkaline samples. A significant difference was also found between As and Pb bioaccessibility in mining residues and mining-affected soil samples. Overall, total Pb an As concentrations and pH were the most significant predictors of Pb and As bioaccessibility. Due to the lack of (quantitative) mineralogical data in many papers, it was not possible to make precise predictions of As and Pb bioaccessibility based on mineralogical sample composition. Despite the challenging nature of quantitative mineralogical characterization, it can contribute to a more precise estimation of the bioavailability of Pb and As in mining waste. Given their significant impact on the bioavailability of metal(loid)s, pH and the (quantitative) mineralogical sample composition should be more systematically determined and reported.

## 1. Introduction

The mining industry is an important industrial sector, as numerous industries rely upon the supply of metals and minerals that are only provided through mining processes. However, the mining industry is also one of the most polluting sectors due to the production of mining waste, acid mine drainage, and land scape degradation. Mining waste can be defined as “solid, liquid, or gaseous by-products of mining, mineral processing, and metallurgical extraction” [1]. These by-products are materials such as topsoil overburden, waste rock, and tailings that accumulate at mine sites. They often contain hazardous substances, such as metal(loid)s, and may generate acid or alkaline drainage [2].

### 1.1. Mining Residues

During almost all mining process steps and throughout the whole life cycle of the mine, from exploration to closure, there is a constant generation of residues, usually considered “waste”, although useful applications are often possible. Three types of mining waste stand out because of their larger volume: waste rock, tailings, and slags [1]. Waste rock is generated during the exploitation step by removing the heterogeneous material that lays between the desired metal or mineral and the workplace. The amount of waste rock that needs to be removed depends on the location of the ore as well as the mining method. Waste rock is often deposited in piles and has a variable chemical and mineralogical composition, depending on the local geology and location of the mining site. Tailings consist of a fine-grained slurry with a high water content, generated during the enrichment process of the ore. The amount of tailing materials that is produced depends on the ore grade, which is the proportion of valuable minerals in the ore. Tailings are stored in embankments to prevent leakages to the environment. A third type of waste that is quite common in the mining industry is slag [1]. Slags originate from the pyrometallurgical processing of ores. If managed correctly, it is a useful product as a construction material or for reprocessing for secondary metal recovery. It contains mostly Si dioxide and metal oxides and appears to have a glassy look [3].

### 1.2. Bioaccessibility of Metal(loid)s

In areas affected by mining activities, incidental oral ingestion and/or inhalation of very fine mining waste particles is a significant exposure way to metal(loid)s such as Pb and As. Crawling and hand-to-mouth behaviour make oral ingestion of particles (soil, dust, waste, etc.) a dominant non-dietary exposure route of children to pollutants, such as metal(loid)s [4]. Geophagy, the practice of consuming clay or soil, is commonly found among pregnant and lactating women in sub-Saharan Africa [5]. Geophagy exposes consumers to metal(loid)s, which can be toxic, depending on their concentration and speciation [6,7]. However, compared to incidental exposure to mining waste particles, geophagy is a much less significant way of exposure. The actual health risk following ingestion of particles depends on the bioaccessibility and bioavailability of the metal(loid)s. Moreover, the absorption of metalloids also varies with the physiology of the exposed person, fasting or age, and the presence of other elements such as Fe and Ca [6].

The health impact of human exposure to hazardous substances can be assessed in different ways, including biomonitoring studies, in vivo testing, model prediction, in vitro tests, etc. In vivo tests study the bioavailability of elements in solid materials by using living organisms. These in vivo tests, however, are expensive and receive some backlash because of ethical concerns. Therefore, in vitro tests are a less controversial and more economical option, and they have become widely used. In vitro bioavailability tests mimic gastrointestinal or pulmonal conditions to study the behaviour of certain elements in the human body. Elements can be taken up in various ways, through ingestion, inhalation, and dermal contact. Absorption through ingestion entails exposure via the mouth, esophagus, stomach, small intestine, and liver [8]. Oral in vitro tests mimic the gastrointestinal tract and in most cases include two main stages: the gastric stage and the intestinal stage. In some tests, the gastric and intestinal phase are preceded by a phase representing the action of saliva in the oral cavity [8,9]. During the gastric stage, the conditions (pH, temperature, enzymes) in the stomach are mimicked, while in the intestinal stage, these conditions are adjusted to the small intestine.

In the literature, the terms, bioavailability and bioaccessibility, are often used simultaneously even though they have a slight different meaning. Bioavailability refers to the amount of a contaminant that is absorbed into the bloodstream and subsequently redistributed throughout the body [10]. The absorbed contaminant can in consequence cause a biological response, be it short- or long-term [11]. The dose–response relationship traditionally measures the effect of an external substance on an organism. The bioavailability of an element in soils or other matrices indicates the availability of a certain element to enter into a living organism and be absorbed by it [12]. Bioaccessibility, on the other hand, is the amount of a contaminant that is present in the body in an organ like the gastro-intestinal tract but not yet absorbed into the blood stream [10]. The in vitro methods addressed in the present study test for bioaccessibility.

### 1.3. Problem Identification and Objectives

Over the past decades, many papers have been devoted to the bioaccessibility of lead and arsenic in mining-affected soils and mine waste, generating many data. However, every mining-affected soil and mining waste has particular characteristics depending on its origin and physical, mineralogical, and chemical properties such as pH, grain size distribution, mineralogy, elemental composition, etc. [12]. It is not always clear how these factors influence the in vitro bioaccessibility of Pb and As in mining waste and mining-affected soils.

The aim of the present paper is to investigate, by using published data, how bioaccessibility of As and Pb is affected by the parameters mentioned above. Additionally, this meta-analysis should also reveal whether significant differences in bioaccessibility of Pb and As exist between various types of mining waste and mining-affected soils. Lastly, we also want to assess whether different bioaccessibility test protocols give significantly different results for the oral bioaccessibility of Pb and As in mining waste and mining-affected soils.

## 2. Materials and Methods

A quantitative meta-analysis of data on the bioaccessibility of lead and arsenic in mining waste and mining-affected soils was performed by combining and analyzing the data from various studies, thereby following consecutive steps. First, by searching multiple databases and identifying, screening, and reviewing publications, relevant publications were retrieved. Secondly, from the selected publications quantitative and qualitative data were extracted. Thirdly, a statistical analysis of the data was performed.

### 2.1. Literature Review and Selection of Relevant Papers

The literature search was performed by using multiple databases including Google Scholar, Web of Science, Elsevier Science Direct, PubMed, and Springer Link. The key words for the literature search were “arsenic” OR “lead” AND “oral” AND “bioavailability” OR “bioaccessibility” AND “in vitro” AND “mining waste” OR “mine” OR “mining”. To find more eligible publications and to complete information, the snowball search method was used as well as the citation search method. Hereby, the relevant cited publications and citing publications bring together different points of view and verify the quality of the information collected.

For the literature screening process, the PRISMA 2020 flowchart [13] was used. This tool aids in minimizing bias and errors and provides a comprehensive methodology for a systematic literature review.

Some inclusion and exclusion criteria were applied to further identify and determine the eligible publications. Only publications that used an in vitro method to determine the oral bioavailability of lead or arsenic were included. It was also important to exclude studies that performed these tests on soils not affected by mining activities, or on ‘waste materials’ other than mining waste. This narrows the search greatly, as a significant number of studies were conducted by using soils affected by agricultural or industrial pollution. Publications using spiked soils were also excluded, as well as publications that did not examine lead or arsenic.

All searches were performed in English, and the year of publications was between 1996 and 2023. Nineteen nighty-six was chosen as a starting year because this is the year Ruby et al. [14] published an article in which the in vitro method PBET (physiological-based extraction test) to predict the bioavailability of metals from a solid matrix was applied for the first time. Since then, several adjustments have been made to this method, and other bioaccessibility tests have been developed.

**Figure 1:**
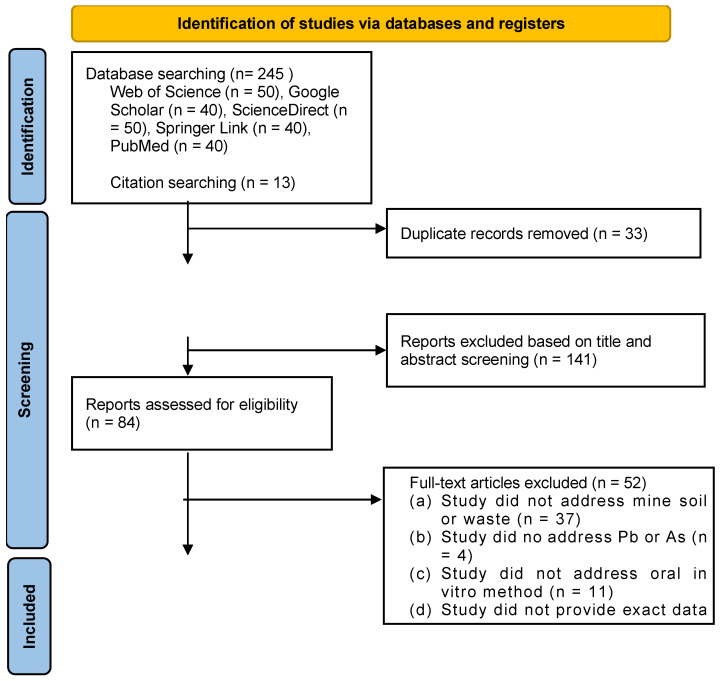
PRISMA flowchart (Criteria only applicable for data extraction).

The initial search resulted in a collection of 258 articles. The subsequent selection took place in various steps, starting with the removal of duplicates followed by exclusion of articles based on title and abstract screening. The next step used the inclusion and exclusion criteria as guideline to screen for eligible articles. Based on these eligibility criteria, a total of 32 articles were included in the literature review (Figure 1), of which 23 articles were used to retrieve data for the statistical analysis. We want to stress that, besides the articles mentioned in Table 1, there are several good publications on the bioaccessibility of As and Pb in mining waste and mining-affected soils. However, the data on bioaccessible As and Pb concentrations are not always provided, which means they cannot be used in the analysis. For example, Xie et al. [15] mentioned that the data are confidential. The data of Bari et al. [16] were not included in the statistical analysis, since bioaccessibility was provided for three different grain size fractions, but the grain size distribution for each sample was not provided. In other studies (e.g., Ettler et al. [17]), no results for individual samples are provided, but basic statistics (average, minimum, maximum, etc.) of all results together.

### 2.2. Data Extraction

From the 23 selected articles, the data were retrieved and collected into an Excel spreadsheet. After a general screening of the article text, the articles were read in more detail, focusing on the methods and results. Through the help of the highlighting function in Zotero, additional important information was collected and put into the data table.

Each row in the Excel file contains the information of one sample, meaning that several rows were created per article. The information collected about each sample consists of the following: type of in vitro method, sampling location, type of mine, type of sample, pH, total concentration of As and Pb in mg/kg, gastric and intestinal bioaccessibility of As and Pb in mg/kg, soil organic matter content, clay content, sand content, silt content, total organic carbon, cation exchange capacity, and mineralogy. If data were only given in graphs, the data presented graphically were extracted with Plotdigitizer (https://plotdigitizer.com/app (accessed on 24 February 2024). We acknowledge that digitized data might be an additional source of uncertainty, but it did allow the dataset to be supplemented. Additionally, the sample name as given in the article, as well as the names of the authors and the year of publication, were included to make it easier to retrieve the original article if needed.

### 2.3. Data Processing

#### 2.3.1. Data Clean-Up

The information in the data table was reviewed in the programming software R version 4.3.3 and R Studio (version 2024.04.0), with the help of AI tool ChatGPT 3.5. All categorical variables were explored and adjusted as needed. The variable “In Vitro Method” contained some double expressions of identical methods, e.g., “PBET,IVG” and “PBET/IVG”, which were merged. The same goes for the variable type of sample, e.g., “mine waste” and “mining waste”. The variables SOM (soil organic matter) and TOC (total organic carbon) were merged together into the variable “OM” (organic matter) using a conversion factor of 0.58 [18].

#### 2.3.2. Statistical Data Analysis

Statistical analysis was performed with the software package R 4.3.3 and R Studio (2024.04.0). All numerical variables were first checked for normality. By creating a quantile–quantile plot and boxplot, the normal distribution was checked visually, while the Shapiro–Wilk test was also performed. Outliers were detected, using the interquartile range, and removed. The linear relationship between two variables was evaluated with a scatter plot.

Descriptive statistics (median, minimum, maximum) were calculated for each variable. Unpaired Welch’s two-sample *t*-tests and ANOVA (Analysis of Variance Analysis) were performed to test equality of samples. Welch’s *t*-test was preferred over a pooled *t*-test as it does not assume equal sample sizes. Correlations between variables were tested by calculating two-tailed Pearson’s correlation for the log_10_-transformed values. Pearson’s correlation was preferred over Spearman’s as the goal was to analyze the linear relationship in the normally distributed data that contains continuous variables.

To assess the appropriateness of combining data from different studies and assure reliability of the results, a heterogeneity test was performed. For each variable (total As and Pb concentration, pH, organic matter content, and clay, sand, and silt content), the correlation coefficients with gastric and intestinal As and Pb bioaccessibility were calculated per article and transformed into Fisher’s Z values using Fisher’s Z-transformation [19]. Higgins’ I_2_ statistic was applied to test for heterogeneity, as it was recommended by Lin et al. [20]. The following rule of thumb was used: I_2_ > 85% indicates high heterogeneity. The results of the heterogeneity test (Supplementary Materials, Table S1) showed that for total concentrations of As and Pb, fairly high but acceptable heterogeneity was found. The variables sand and silt content were not eligible for further analysis due to small sample size (since sand and silt content was not determined in all papers). The variables pH and organic matter content showed low heterogeneity and could be included, while clay content showed a high heterogeneity and was excluded from further analysis except for gastric As bioaccessibility (I_2_ = 70.6%).

Principal component analysis (PCA) was performed to gain a better insight in the relationship between variables. It is widely used for the evaluation of environmental data [21,22]. A scree plot was used to visually determine the optimal number of components or factors to retain, ensuring that the eigenvalues of the used components were above 1. ANOVA was used to determine whether differences exist between different types of samples and between different in vitro bioaccessibility tests. Tukey’s Honestly Significant Difference (HSD) post hoc test was used to determine which groups are significantly different from each other.

A stepwise multiple linear regression was performed to estimate the relationship between bioaccessibility as a dependent variable and soil/waste material properties as independent variables. Independent variables were organic matter, clay, sand, silt content, and total concentrations of Pb and As, with the dependent variables being gastric and intestinal bioaccessibility of Pb and As. Gastric bioaccessibility was included as independent variable for the regression model of intestinal bioaccessibility. The regression was performed according to the stepwise method in order to include the most significant independent variables. Stepwise regression was performed using the backward elimination method. The AIC (Akaike information criterion) decision criterium was used by default [23].

A scatter plot of residuals versus predicted values confirmed homoscedasticity. To test for multicollinearity, the Variance Inflation Factor (VIF) was calculated, and the general rule of thumb that a VIF of 10 or greater is a cause for concern was followed [23].

## 3. Results and Discussion

In this section, we first give an overview of the literature screening process and the data retrieved from 23 publications. Then, the data are subjected to a statistical data analysis to investigate whether (1) the sample type and the sample composition influence the bioaccesssibility of As and Pb, (2) how sample properties can be used to predict the bioaccessibility of Pb and As, and (3) whether there is a significant difference between different protocols for in vitro bioaccessibility.

### 3.1. Literature Data on Bioaccessibility of As and Pb in Mining Waste and Mining-Affected Soil

An overview of the literature screening process is given in Figure 1 using the PRISMA 2020 flowchart [13]. From the 245 articles that were initially retrieved, 33 duplicates were removed, leaving 225 articles for screening based on abstract and title. This initial screening resulted in 84 articles, of which 52 were excluded during full-text screening for various reasons: 37 articles did not involve mine waste or mining-affected soil, 4 did not analyze Pb or As, and 11 articles did not use an oral in vitro method. Thirty-two articles were thus relevant for this study. However, only 23 articles were included, as 9 articles did not provide the exact data required. In total, data from 228 samples were retrieved from 23 articles (Table 1).

The majority of the samples came from Europe (n = 94), followed by the Americas (Canada, Brazil, and Cuba) (n = 52). Most samples (n = 56) originated from mine tailings, while only a few samples were labelled as waste rock (n = 7) or slag (n = 11). When the type of mine waste was not specified in the paper, the general term ‘mine waste’ was used (n = 53). Additionally, 101 samples published in 15 different articles were labelled as ‘soil’.

**Table 1:** Summary of data sources, location and type of samples, and type of in vitro method used. n = number of samples.

	Year	n	Location	Sample Type	In Vitro Method
[24]	2006	18	Spain	Slag, tailing, urban soil	SBRC
[25]	2007	7	Australia	Mine waste, soil, waste rock	PBET
[26]	2007	8	Australia	Soil	PBET
[27]	2008	12	Brazil	Mine waste, slag, soil	PBET
[28]	2011	2	Australia	Soil	UBM
[10]	2011	9	Turkey	Soil	PBET
[29]	2011	27	Canada	Tailing	PBET
[30]	2012	2	China	Soil, urban soil	IVG
[31]	2012	9	Brazil	Soil, tailing	IVG
[32]	2013	42	Wales	Mine waste, tailing	PBET
[33]	2013	4	Cuba	Tailing	IVG
[34]	2014	6	China	Soil, tailing, waste rock	PBET
[35]	2014	4	France	Mine waste, soil, tailing	UBM
[36]	2015	4	China	Soil,	SBET
[37]	2016	6	Australia	Mine waste, tailing	SBRC
[38]	2017	18	Nigeria	Soil	RBALP
[39]	2018	11	Czech Republic	Mine waste, urban soil	SBRC
[40]	2018	3	China	Soil	PBET
[41]	2019	6	Australia	Slag, soil	UBM
[42]	2019	11	China	Soil	UBM
[43]	2020	4	Belgium	Mine waste	PBET
[44]	2020	8	Italy	Soil, tailing, waste rock	UBM
[45]	2022	7	Portugal, Germany, Belgium	Mine waste, tailing, waste rock	PBET

The type of in vitro methods used to assess the bioaccessibility of lead or arsenic is presented in Table 1. Almost 55% of the samples (n = 125) were subjected to the PBET, followed by SBET (Simplified Bioaccessibility Extraction Test) and SBRC (Solubility Bioaccessibility Research Consortium), which were applied on 39 samples (from four publications). The other in vitro bioaccessibility tests were applied on less than 10% of the samples.

A total of 8 out of 23 articles included data on the mineralogical composition of the samples, representing 90 samples out of 228. Of these 8 articles, only 3 articles provided quantitative mineralogical information, representing 57 samples [32,43,45]. Quartz occurs in 53 out of 57 samples, with a median concentration of 49.4%. Arseniosiderite (Ca_2_Fe^3+^ _3_(AsO_4_)_3_O_2_·3H_2_O) was found in five samples from the dataset, with median value of 57%. Cerussite (PbCO_3_) and anglesite (PbSO_4_) were the most commonly reported Pb minerals (in resp. 19 and 22 samples) (Supplementary Materials, Figures S1 and S2). Overall (for samples for which qualitative and quantitative data are provided), quartz, amorphous phases, feldspars, and clay minerals were the most commonly reported matrix minerals, followed by goethite (FeO(OH) and pyrite (FeS_2_). Other Pb minerals were lanarkite (Pb_2_(SO_4_)O) and galena (PbS). The arsenic-bearing minerals in the samples consist of arseniosiderite (Ca_2_Fe^3+^ _3_(AsO_4_)_3_O_2_·3H_2_O), jarosite (KFe^3+^ _3_(SO_4_)_2_(OH)_6_), arsenopyrite (FeAsS), beudantite (PbFe^3+^ _3_(AsO_4_)(SO_4_)(OH)_6_), loellingite (FeAs_2_), and realgar (As_4_S_4_).

The variable “pH”, which has an important influence on the mobility of metal(loid)s in soil and waste materials [46], showed a bimodal distribution (Supplementary Materials, Figure S3). Therefore, for some statistical analyses the dataset was divided into two groups of samples with ‘acidic pH’ and ‘neutral to alkaline pH’ according to the separation point, which was calculated at 5.63 by selecting the median value between the two peaks. Group 1 (pH < 5.6) and Group 2 (pH ≥ 5.6) contained 58 and 90 samples, respectively. For the multiple linear regression analysis, data from six articles were excluded as they did not provide data on pH. Since the pH value of 5.6 is statistically derived, and its geochemical significance is not straightforward, the regression was also conducted for the complete dataset (including both groups). With respect to the particle size of the investigated samples, it is clear that there is no consensus on which particle size to investigate in bioaccessibility studies. Some researchers use a threshold of less than 150 μm [27,29,40], while others opt for less than 250 μm [24,41,44]. Both groups use the same explanation: particle size is believed to most likely stick to human hands and be ingested through hand-to-mouth or pica activities [31]. Some studies found that particle size variations appeared to be insignificant in explaining the variability of As bioaccessibility in abandoned mine soils [16]. In contrast, Smith et al. [47] concluded that arsenic bioaccessibility increased with decreasing particle size in soils contaminated with As through historical agricultural or industrial sources. Li et al. [48] demonstrated that there is no clear correlation between particle size distribution and the bioavailability of metal(loid)s in soils, and that bioaccessibility was more related to the source and to the physico-chemical form of metal(loid)s [48].

Table 2 gives a general overview of the total and bioaccessible concentrations of As and Pb, grain size distribution in the sample, and organic matter content for samples with an acidic pH (pH < 5.6) and samples with a neutral to alkaline range (pH ≥ 5.6).

Total Pb concentrations were significantly lower in samples with a neutral or alkaline pH with a mean of 11,154 mg/kg compared to 72,119 mg/kg for samples with an acidic pH. The organic matter content was 0.24–46.6%, while the clay content was in the range of 2.0–53.5%. The silt and sand content exhibited a range of 5.3–71% and 2.33–86.3%, respectively.

The distribution of gastric and intestinal bioaccessible Pb and As concentrations is right-skewed with a long tail (Supplementary Materials, Figure S4). The range of gastric bioaccessible As concentrations is therefore very broad, from 0.17 to 40,002 mg/kg. The intestinal bioaccessibility of As has a lower density in the first quartile, with concentrations ranging from 0.078 to 4296 mg/kg and a median value of 28.71 mg/kg. Similar observations can be made for Pb, with the exception that the density is even lower.

### 3.2. Influence of Sample Type and Physico-Chemical Sample Characteristics on Bioaccessibility

Total and bioaccessible concentrations of Pb and/or As were the only data available for all the samples. For this dataset, a Pearson’s correlation analysis was performed, showing that gastric and intestinal bioaccessible As and Pb were positively correlated (*p* < 0.01, 95% confidence level) with, respectively, the total concentration of As and Pb (Table 3).

Data on grain size distribution (clay, silt, and sand content) and organic matter content were not available for all samples; so, correlation coefficients only apply to a part of the dataset. Tables S2 and S3 (Supplementary Materials) present the Pearson’s correlation matrix for acidic samples (pH < 5.6) and neutral/alkaline samples (pH ≥ 5.6) separately.

The pH demonstrated a positive correlation with gastric Pb bioaccessibility (r = 0.410) in acidic samples (pH < 5.6), while in neutral and alkaline samples (pH ≥ 5.6), there was a negative correlation (r = −0.400). The clay content was found to be negatively correlated with the total Pb concentration (r = −0.724) and positively with gastric As bioaccessibility (r = 0.642) in the acidic samples, while there was no significant correlation for the neutral and alkaline samples. Cui et al. [49] reported that the bioaccessibility of Pb and Cu in gastric and intestinal phases decreased by 0.4 to 6% with every unit increase in soil pH.

In the present study, gastric bioaccessibility of As was significantly higher than interstinal bioaccessibility (Table 2). Several studies find that arsenic bioaccessibility in soil is higher in the gastric phase because the low pH facilitates the dissolution of arsenic-bearing minerals. In the intestinal phase, the increase in pH to near-neutral levels can cause arsenic to re-adsorb onto precipitated iron oxides, sometimes leading to a decrease in measured bioaccessibility [50].

The human gastrointestinal tract is a highly dynamic environment with a steep redox gradient, transitioning from a strongly oxidizing environment in the stomach to a highly reducing environment in the large intestine [51]. Redox conditions alter the chemical speciation of arsenic and its interaction with soil and mineral matrices, significantly influencing its oral bioaccessibility [52]. The in vitro tests are not performed under controlled redox conditions and therefore do not capture this redox gradient.

Bari et al. [16] also found a significant positive linear correlation between total and bioaccessible As concentrations, both for the gastric phase and intestinal phase of the PBET and SBRC. In contrast, Canovas et al. [53] reported that the correlation between the total and bioaccessible concentrations of Pb and As in sulphidic mine waste samples was very low or simply lacking.

Principal component (PCA) analysis was performed to find a possible influence of the sample type (soil, mine waste, waste rock, slag, and tailing) on bioaccessibility (Supplementary Materials, Figures S5 and S6). PC1 and PC2 account for 53.17% and 57.23% of the variance observed in Figures S5 and S6, respectively, with the variables total Pb concentration (Pb_TOT_) and gastrointestinal Pb bioaccessibility (Pb_IN_) being the most heavily weighted variables on PC1 with a negative loading factor of more than −0.5. For PC2, the highly weighted variables are total As concentration and gastrointestinal As bioaccessibility for both PCAs. There is, however, a strong overlap among the sample types, and thus the impact of sample type on bioaccessibility cannot be obviously discriminated (Supplementary Materials, Figures S5 and S6).

Based on the PCA, there is therefore no strong evidence that the diverse types of samples influence bioaccessibility. In a second step, a one-way ANOVA was performed in order to test the statistical difference in bioaccessibility of Pb and As between the sample types (Table 4).

The one-way ANOVA demonstrated a robust and statistically significant *p*-value at a 95% confidence level for each bioaccessibility group, i.e., Pb_G_, Pb_IN_, As_G_, and As_IN_, indicating that there is a difference in gastric and intestinal bioaccessibility of Pb and As across sample types. Tukey’s post hoc test indicates which differences are statistically significant (Table 4). In the majority of cases, the sample type “soil” is significantly different from the sample types “mine waste, waste rock, slag or tailing”. A distinction can therefore be made between two sample groups: mining residue (including mine waste, tailing, slag, and waste rock) and soil. The two sample groups are indeed significantly different from each other, except for the gastric bioaccessibility of Pb.

For the further analysis, a distinction was therefore made between two sample groups: mining residue (including mine waste, tailing, slag, and waste rock) and soil. From now on, the term ‘mining residue’ will thus refer to mine waste, waste rock, slag, and tailing, which are considered one group of samples.

A stepwise multiple linear regression was initially performed on all data, followed by separate regressions on “mining residue” and “soil” samples (Table 5). With this, the influence of total As and Pb concentration and sample properties, including pH, organic matter, and clay content, on As and Pb bioaccessibility was investigated. Total As and Pb concentrations, as well as pH, proved to be significant explanatory variables for the gastric bioaccessibility of resp. As and Pb for the whole dataset, as well as for “mining residues” and soils” analyzed separately. Organic matter and clay content were not retained as independent variables. Intestinal bioaccessibility was dependent on total concentration, pH, and the concentration of As or Pb already removed during the gastric phase.

Stepwise linear regression was also performed on the “acidic” and “neutral to alkaline” sample groups separately (Supplementary Materials, Table S4). For the ‘acidic’ samples (mining residues and soil together = ‘all’), as well as for the alkaline to neutral soil samples, gastric As bioaccessibility was explained by the dependent variables “total As concentration” and “organic matter content”. However, organic matter content was only available for a limited amount of samples (resp. for 19 and 24 “acidic samples” and “neutral to alkaline” soil samples). The clay content was an explanatory variable for gastric As bioaccessibility in acidic soils and in alkaline mining residues. However, the subdivision of the data by pH group, in addition to the subdivision by sample type sometimes results in small datasets for which the regression equations often, have low significance.

In general, there was no significant increase in R^2^ when acidic and ‘neutral to alkaline’ samples were considered separately, compared to the overall data set. This corroborates the previous assertion that there is no strong evidence to suggest that the bioaccessibility is significantly different in the acidic versus neutral–alkaline samples. One exception is the gastric As and Pb bioaccessibility of samples in the neutral and alkaline group, where the regression equations for the mining residue have a higher coefficient of determination (R^2^) (Table S4), compared to the regression equations for the complete dataset (Table 5).

We are well aware that the dataset used in this study is heterogeneous. Generalizing results from heterogeneous datasets is difficult because these variations introduce biases and inconsistencies that a single model cannot accurately represent.

### 3.3. Influence of Mineralogical Composition on Bioaccessibility of Pb and As

The original intention was to investigate the influence of mineralogical composition on the bioavailability of As and Pb. Given the very limited amount quantitative mineralogical data in scientific papers, this section is limited to a more qualitative discussion.

Bioaccessibility studies use a variety of methods such as X ray diffraction, SEM-EDS, XAFS, FEG-EPMA, etc., for a mineralogical and morphological characterization of the samples. Some studies addressed the bioaccessibility of pure minerals to understand how the minerals are affected by the conditions in the digestive tract [54] (Table 6). Since a quantitative approach is not straightforward, the information on bioaccessibility of minerals found in the literature was qualitatively described from very low to high bioaccessibility (Table 6).

Base metal sulfides such as galena, chalcopyrite, and sphalerite were not abundant in the samples, with median concentrations of 1.1%; 5%, and 0.95%, respectively, but they can carry the majority of metal(loid)s present in waste. The minerals hematite (Fe_2_O_3_) and magnetite (Fe_3_O_4_) were also present and result from the transformation of arsenopyrite and pyrite, both of which are iron sulfide minerals [55]. At mining sites, lead minerals can be encapsulated by quartz, which might influence its bioaccessibility [56,57]. Bioaccessibility of As and other metal(loid)s in soil and mine waste is also influenced by the retention capacity of the soil or mining waste under gastrointestinal conditions [49].

**Table 6:** Qualitative assessment of the bioaccessibility of Pb and As minerals, based on the bioaccessibility of pure minerals or on studies that performed a quantitative mineralogical characterization.

	Test	Result	Ref.
Pb-minerals			
cerrusite	SBET	High in vitro bioaccessibility	[58]
	PBET	High in vitro bioaccessibility	[45]
	PBET	High in vitro bioaccessibility	[32]
	In vivo	High in vivo bioaccessibility	[59]
	In vivo	High in vivo bioaccessibility	[60]
galena	SBET	Low bioaccessibility	[58]
	In vivo	Medium in vivo bioaccessibility	[59]
	SBET	Low in vitro bioaccessibility	[17]
Pb-oxides	PBET	Higher in vitro bioaccessibility than Pb-phosphates, -chromates, and -sulfates	[61]
Pb-goethite	UBM	Partly dissolved	[62]
	0.07 M HCl	Partly dissolved	[63]
anglesite	0.07 M HCl	Not partially dissolved	[63]
	UBM	Partly dissolved	[62]
	SBET	Low bioaccessibility	[58]
As-minerals			
arseniosiderite	-	High relative bioaccessibility	[55]
	SBET	Medium in vitro bioaccessibility (6–12%)	[54]
	PBET	Higher bioaccessibility then arseniosiderite	[64]
arsenopyrite	PBET	Low bioaccessibility	[65]
scorodite	SBET	Almost insoluble	[63]
	SBET	Very low bioaccessibility (0.13%)	[64]
amorphous Fe-arsenates and As-bearing Fe-(oxy)hydroxides	PBET	Medium bioaccessibility (1–10%)	[65]
	SBRC	Low bioaccessibility	[66]

### 3.4. Comparison of In Vitro Methods to Determine Bioaccessibility in Mining Waste

Different oral in vitro bioavailability methods, which have been applied to mining waste and mining-affected soils, were compared. This paper only considers oral bioaccessibility and not the exposure via inhalation (pulmonal bioaccessibility) or dermal contact. In recent decades, several protocols for in vitro methods have been established. Six different protocols have been reported in the research papers included in the present study: Physiological Based Extraction Test (PBET), Unified BARGE Method (UBM), In vitro Gastrointestinal method (IVG), Rijksinstituut voor Volksgezondheid en Milieu (RIVM), Solubility Bioaccessibility Research Consortium method (SBRC), and Relative Bioaccessibility Leaching Procedure (RBALP). The six methods consist of comparable steps, with differences with respect to reagents used, liquid/solid ratios, reaction times, ways of agitation, fluid separation, filtration, and analysis (Supplementary Materials, Table S5). All the methods operate at a temperature of 37 °C, and the amount of samples used for the tests ranges from 0.06 g to 4 g.

The Physiological Based Extraction Test (PBET) was first developed by Ruby et al. [14] and is a method that simulates the leaching of a solid matrix in the human gastrointestinal system, considering a gastric phase (using pepsin together with a mixture of pancreatin, amylase) and an intestinal phase (using bile salts) [10]. The IVG method, which is comparable with the PBET, was developed by Rodriguez et al. [67] to design a good predictor for arsenic bioavailability.

The Unified BARGE Method (UBM) distinguishes three different stages in the human digestive system: oral cavity (using saliva), the gastric stage (using a gastric fluid), and the intestinal stage (using duodenal fluid and bile). BARGE is the Bioaccessibility Research Group of Europe [9]. In 2018, the International Organization for Standardization released a standardized method for the assessment of human exposure from ingestion of soil and soil material, specifically for the estimation of the human bioaccessibility/bioavailability of metals in soil [68]. The method is based on the UBM method. The RIVM method, originally from the Netherlands, is also very similar to the UBM model.

The SBRC and RBALP, which have been developed to assess Pb bioaccessibility (reference), are very similar to each other for what concerns the gastric phase. However, the RBALP method does not integrate an intestinal phase and is thus a non-physiologically based method that is seen as the most cost-effective and fastest method [57].

A one-way ANOVA analysis was performed to statistically test whether or not a distinction can be made for the different in vitro methods, based on the data on As and Pb bioaccessibility used in this paper (Table 7).

The ANOVA analysis demonstrated that there is a difference in relative bioaccessibility (i.e., bioaccessibility relative to total concentration of Pb and As) outcomes (with the exception of Pb intestinal) across different in vitro methods. To verify which in vitro methods were mutually different, a post hoc test was conducted. The results from the in vitro method SBRC were significantly different from the PBET for gastric Pb and As bioaccessibility and for intestinal As bioaccessibility. Several authors compared different bioaccessibility tests. A study by Cao et al. [41] showed that As bioaccessibility varied depending on the in vitro method used (SBRC, IVG, PBET). Meunier et al. [69] and Bari et al. [16] compared As bioaccessibility with the SBR and PBET protocols and found that bioaccessible As in the intestinal phase of PBET and SBRC was significantly higher than in the intestinal phase of SBRC, most likely caused by the buffer in the SBRC method. Results of different bioaccessibility tests are often not comparable due to, among others, the physiological compartments included as well as the residence time in each compartment, the pH of each compartment, the composition of the digestive fluids, the liquid-to-solid ratio, etc. [70]. The use of different grain size fractions might also have a significant influence on the results [16].

## 4. Conclusions

This study investigated the correlations between sample (soil and mining residue) physicochemical properties and As and Pb gastric and intestinal oral bioaccessibility. Overall, total Pb an As concentrations and pH were the most significant predictors of Pb and As bioaccessibility.

The results showed that mean gastric As bioaccessibility was lower (*p* < 0.001) in acidic samples than in neutral and alkaline samples. A significant difference was also found between As and Pb bioaccessibility in mining residue and mining-affected soil samples. The bioaccessibility of As and Pb determined with the in vitro methods SBRC and PBET was significantly different, both in mining waste and soils. The many different protocols for bioaccessibility testing hinder the comparison between different studies due to the inconsistent use of particle size fractions for the bioaccessibility tests, different incubation times, different liquid-to-solid ratios, etc.

Moreover, the lack of standardization in reporting data from bioaccessibility test by different studies is a major challenge. To be transparent, data reporting should meet quality requirements, which should also be specified in the protocols for bioavailability testing. The use of certified reference samples should also be an inherent part of this. Unlike other chemical extraction protocols, such as sequential extractions, the use of reference materials for bioavailability testing is not yet well established. This may be due to the very limited availability of reference materials for bioavailability testing. Furthermore, these materials are usually soil samples, while bioavailability is also relevant for other fine-grained materials such as mine waste and dust. The present study focused on Pb and As, two elements for which bioavailability in mining residues and soils is relatively often investigated. A similar approach could be envisaged for other metal(loid)s, as well as other types of soils and waste materials.

Another limitation is the lack of studies that consider the mineralogical composition of mining-affected soils and mining waste. Surprisingly, in only 8 of the 23 papers (representing 90 samples), information on the mineralogical composition of the samples was provided, and very few papers provided quantitative mineralogical information. It was therefore not possible to make reliable predictions of As and Pb bioaccessibility based on mineralogical sample composition. It is strongly recommended, in the context of bioavailability testing, to quantify the mineralogical composition of materials for which this is relevant and to share data in a more transparent way. Another limitation is the lack of information on As speciation, as different species are characterized by different toxicities, mobilities, and bioaccessibilities.

In mining waste, a high proportion of metal(loid)s is often retained in mineral phases. Some minerals hosting high concentrations of metal(loid)s are characterized by a higher solubility by digestive fluids and a significantly higher bioaccessibility of the metal(loid)s of concern. This highlights the need for a more systematic exploration of mineralogical data, as mineralogy plays an important role in providing a first indication of bioaccessibility.

## Figures and Tables

**Table 2:** Total and bioaccessible concentrations of As and Pb, physicochemical properties of samples, and significance of two-sided *t*-test for samples with a pH < 5.6 and ≥5.6.

	pH < 5.6 (n = 58)			pH ≥ 5.6 (n = 90)			Difference Between Samples with pH < 5.6 and with pH ≥ 5.6 (Two-Sided *t*-Test)
	Minimum	Mean	Maximum	Minimum	Mean	Maximum	*p*-Value
As_TOT_ (mg/kg)	28	2312.20	4620	8	2352	17,400	*p* = 0.134
As_G_ (mg/kg)	0.72	77.81	1356	0.17	501	9379	*p* < 0.001
As_In_ (mg/kg)	0.18	20.94	212	0.08	48.82	438	*p* = 0.117
Pb_TOT_ (mg/kg)	40.80	72,119	131,227	18	11,154	14,379.80	*p* = 0.017
Pb_G_ (mg/kg)	3.40	2121	10,719	1.85	8431	185,603	*p* = 0.904
Pb_In_ (mg/kg)	11.25	21,403	3864	0.27	1162.16	5309	*p* = 0.172
OM (%)	0.24	2.29	15.72	0.29	15.78	46.60	*p* < 0.001
Clay (%)	2.30	19	53.72	2	13.50	37	*p* = 0.428
Silt (%)	5.30	36.76	65.40	17.20	48.58	71	*p* = 0.260
Sand (%)	2.33	46.25	86.30	4.01	35.99	69.60	*p* = 0.065

**Table 3:** Pearson’s correlations between bioaccessible As and Pb (mg/kg) and total As and Pb (mg/kg) of all samples (n = 155 for As, n = 171 for Pb, ** *p* < 0.01).

	AS_TOT_	AS_G_	AS_IN_		PB_TOT_	PB_G_	PB_IN_
AS_TOT_	1			Pb_TOT_	1		
AS_G_	0.687 **	1		Pb_G_	0.899 **	1	
AS_IN_	0.775 **	0.859 **	1	Pb_In_	0.707 **	0.775 **	1

**Table 4:** ANOVA and post hoc test of bioaccessibility in different sample types.

One-Way Anova		Tukey’s HSD Test		
Relative Bioaccessibility	*p*-Value	Type of Sample	*p*-Value	Mining Residue vs. Soil (*p*-Value)
Pb Gastric	*p* < 0.001	soil-mine waste waste rock-mine waste tailing-soil waste rock-tailing	*p* < 0.001 *p* = 0.00175 *p* = 0.01875 *p* = 0.03036	*p* < 0.001
Pb Intestinal	*p* < 0.001	soil-mine waste waste rock-mine waste waste rock-soil waste rock-tailing	*p* = 0.0049 *p* < 0.001 *p* = 0.00219 *p* = 0.02029	*p* = 0.472
As Gastric	*p* < 0.001	soil-slag tailing-slag tailing-soil waste rock-tailing	*p* = 0.088 *p* < 0.001 *p* = 0.00588 *p* < 0.001	*p* = 0.0477
As Intestinal	*p* < 0.001	soil-slag tailing-soil	*p* = 0.00447 *p* = 0.00345	*p* = 0.0099

**Table 5:** Stepwise multiple linear regression analysis, with gastric or intestinal Pb or As bioaccessibility as dependent variables. * *p* = 0.05, ** *p* = 0.001.

	Sample Type	Phase	Regression Model	R^2^
All pH	All (n = 148)	As_G_ (n = 102)	As_G_ = −2.47 + 2.57 * pH + 0.69 * AsTOT	0.3397 **
		As_IN_ (n = 82)	−1.71 + 1.57 * pH + 0.24 * As_TOT_ + 0.63 * As_G_	0.4514 **
		Pb_G_ (n = 123)	−2.41 + 2.2 * pH + 1.23 * Pb_TOT_	0.7931 **
		Pb_IN_ (n = 97)	1.06–0.79 * pH − 0.06 * Pb_TOT_ + 0.73 * Pb_G_	0.5536 **
	Mining residue (n = 91)	As_G_ (n = 50)	−2.98 + 3.37 * pH + 0.67 * As_TOT_	0.308 **
		As_IN_ (n = 49)	−1.85 + 1.46 * pH + 0.35 * As_TOT_ + 0.5 * As_G_	0.4755 **
		Pb_G_ (n = 83)	−3.17 + 2.66 * pH + 1.15 * Pb_TOT_	0.8427 **
		Pb_IN_ (n = 70)	2.29 + 0.41 * pH	0.0022
	Soil (n = 57)	As_G_ (n = 45)	−1.01 + 0.38 * pH + 0.82 * As_TOT_	0.5714 **
		As_IN_ (n = 33)	−0.03–0.04 * pH − 0.16 * As_TOT_ + 1.27 * As_G_	0.5362 **
		Pb_G_ (n = 40)	−0.65 + 0.74 * pH + 0.83 * Pb_TOT_	0.5788 **
		Pb_IN_ (n = 27)	7.05–8.17 * pH − 0.49 * Pb_TOT_ + 1.37 * Pb_G_	0.6404 **

**Table 7:** ANOVA and post hoc test of relative bioaccessibility by six in vitro methods.

One-Way Anova		Tukey’s HSD Test	
Relative Bioaccessibility	*p*-Value	In Vitro Method	*p*-Value
Pb Gastric	*p* < 0.001	RBALP-PBET SBET-PBET SBRC-PBET UBM-PBET UBM-RBALP UBM-SBRC	*p* < 0.001 *p* = 0.00232 *p* < 0.001 *p* = 0.00495 *p* = 0.00214 *p* = 0.04126
Pb Intestinal	*p* = 0.095		
As Gastric	*p* < 0.001	SBRC-PBET UBM-SBRC	*p* < 0.001 *p* = 0.0455
As Intestinal	*p* < 0.001	SBRC-IVG SBRC-PBET UBM-SBRC	*p* = 0.00492 *p* < 0.001 *p* < 0.001

## Data Availability

The original contributions presented in this study are included in the article/Supplementary Materials. Further inquiries can be directed to the corresponding author.

## Supplementary Materials

The following supporting information can be downloaded at https://www.mdpi.com/article/10.3390/toxics14020114/s1. Figure S1: Relative frequency of minerals. Figure S2: Bar plot representing median concentrations of minerals with sample counts. Figure S3: Density plot for pH distribution (n = 148). Figure S4: Density plot of gastric and intestinal Pb and As bioaccessibility. Figure S5: PCA for total As and Pb concentrations and bioaccessibility in five types of samples (pH < 5.6). Figure S6: PCA for total As and Pb concentrations and bioaccessibility in five types of samples (pH ≥ 5.6). Table S1: I_2_ results from heterogeneity test. Table S2: Pearson’s correlation matrix for samples with pH < 5.6 (n = 58). Table S3: Pearson’s correlation matrix for samples with pH ≥ 5.6 (n = 90). Table S4: Stepwise multiple linear regression per sample type and per ‘pH group’. Table S5: Comparison of six in vitro methods.

## Abbreviations

The following abbreviations are used in this manuscript:

AIC	Akaike information criterion
ANOVA	Analysis of Variance
BARGE	Bioaccessibility research group of Europe
HSD	Honestly Significant Difference
IVG	In vitro Gastrointestinal
OM	Organic matter
PBET	Physiological-based extraction test
PCA	Principal component analysis
RBALP	Relative Bioaccessibility Leaching Procedure
RIVM	Rijksinstituut voor Volksgezondheid en Milieu
SBET	Simplified Bioaccessibility extraction test
SBRC	Solubility Bioaccessibility Research Consortium
SOM	Soil organic matter
TOC	Total organic carbon
UBM	Unified BARGE Method
VIF	Variance Inflation Factor
