# Supplementary material for: Bioaccessibility of Lead and Arsenic in Mining Waste and Mining-Affected Soils
Source: Toxics. 2026 Jan 26;14(2):114. doi: 10.3390/toxics14020114 (PMC12944831; doi:10.3390/toxics14020114)
Supplement: Supplementary file 1 [file toxics-14-00114-s001.zip › toxics-4115165-supplementary.pdf]

Supplementary materials

Table S1 I^2 results from heterogeneity test

I^2	As Gastric Bioaccessibility	As Intestinal Bioaccessibility	Pb Gastric Bioaccessibility	Pb Intestinal Bioaccessibility
Total Concentration	81.15%	60.43%	86.36%	79.69%
pH	32.64%	0%	31.96%	0%
Organic Matter	39.42%	0%	34.89%	0%
Clay Content	70.56%	0%	96.18%	88.49%
Sand Content	0%	0%	0%	0%
Silt Content	12.66%	0%	0%	0%

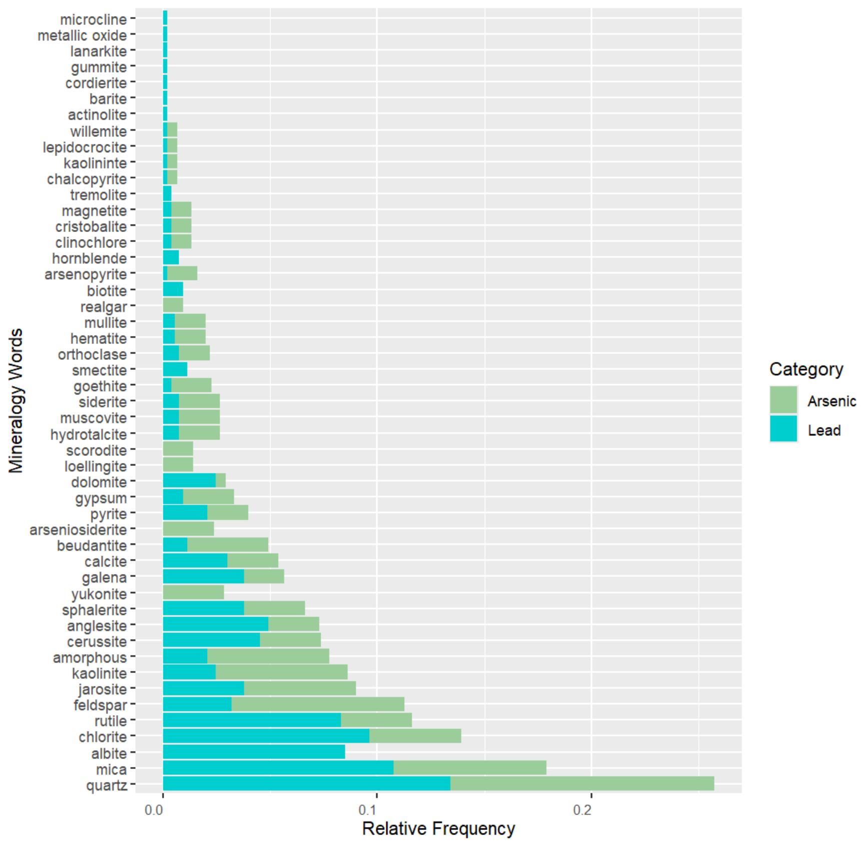

Figure S1 Relative frequency of minerals

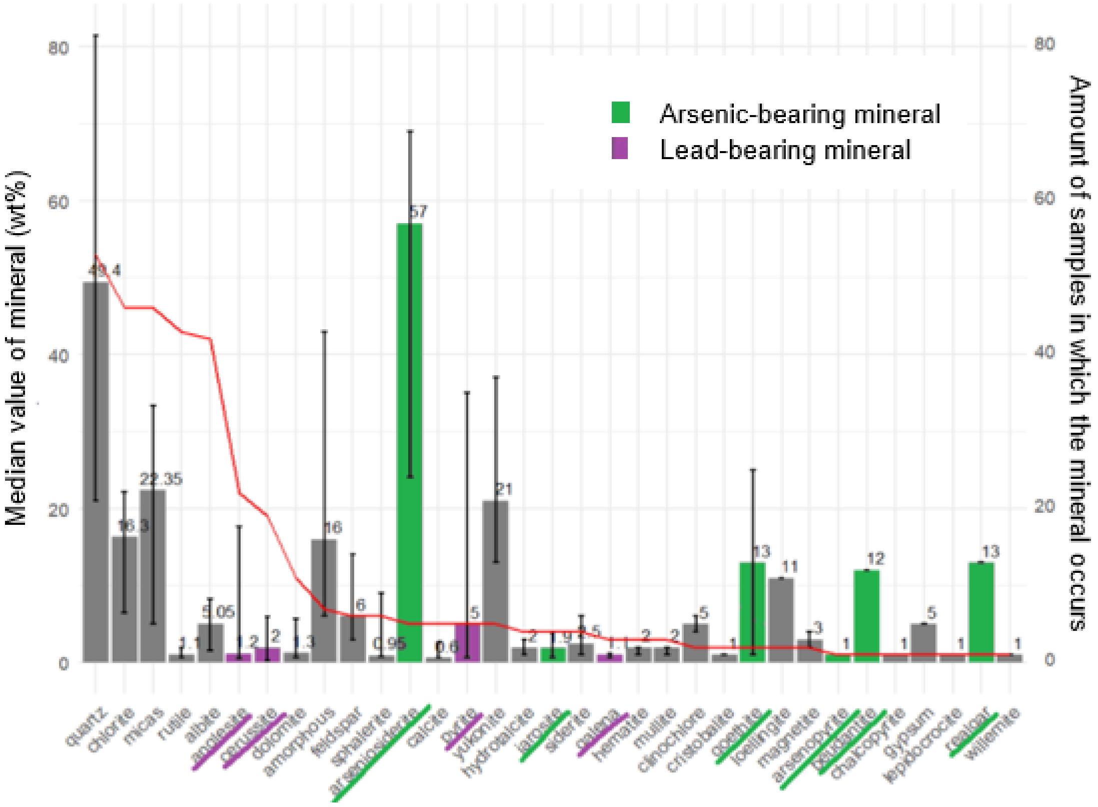

Figure S2 Bar plot representing median concentrations of minerals with sample counts

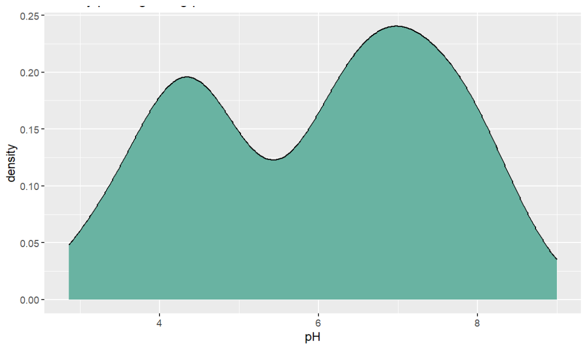

Figure S3 Density plot for pH distribution (n=148)

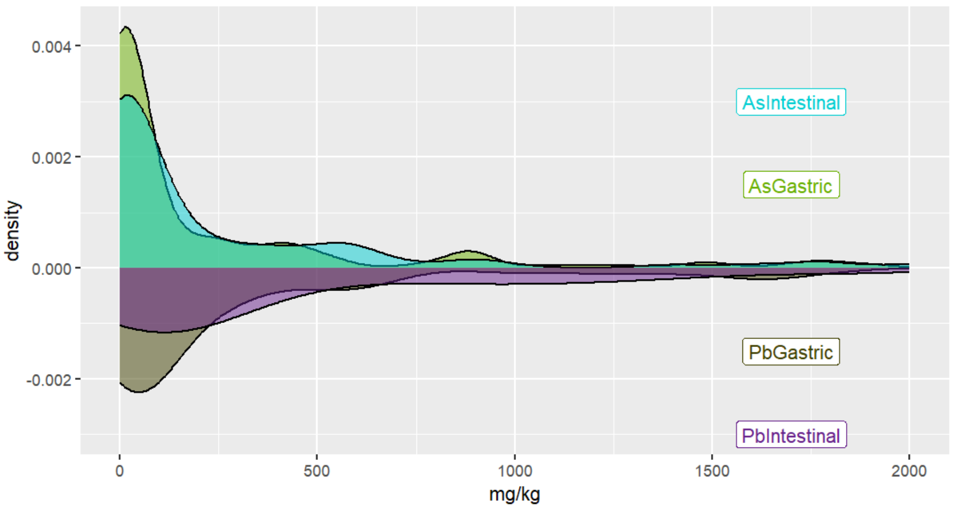

Figure S4 Density plot of gastric and intestinal Pb and As bioaccessibility

Table S2 Pearson correlation matrix for samples with pH<5,6 (n=58)

pH<5,6 (n=58)	**pH**	**OM**	**Silt**	**Sand**	**Clay**	**As_{TOT}**	**Pb_{TOT}**	**As_G**	**As_{IN}**	**Pb_G**	**Pb_{IN}**
**pH**	1										
**OM**	0,092	1									
**Silt**	-0,361	-0,691	1								
**Sand**	-0,173	0,009	-0,614*	1							
**Clay**	-0,214	0,304	-0,365	-0,806**	1						
**As_{TOT}**	-0,049	0,197	-0,121	0,419	-0,250	1					
**Pb_{TOT}**	0,149	-0,122	-0,311	0,769**	-0,724**	0,288	1				
**As_G**	0,126	-0,463	0,150	-0,459[a]	0,642**	0,195	-0,026	1			
**As_{IN}**	0,108	-0,918*	-0,563	0,537	-0,003	0,551*	0,242	0,682**	1		
**Pb_G**	0,410**	-0,310	-0,236	0,226	-0,219	0,260	0,861**	0,150	0,624*	1	
**Pb_{IN}**	0,132	0,257	-0,081	-0,256	0,456	-0,384	0,740**	0,676*	0,323	0,830**	1

[a]p<0,1
*p<0,05
**p<0,01

As_{TOT}= total concentration of arsenic
Pb_{TOT}= total concentration of lead
As_G= gastric bioaccessibility of arsenic

As_{IN}= intestinal bioaccessibility of arsenic
Pb_G= gastric bioaccessibility of lead
Pb_{IN}= intestinal bioaccessibility of lead

Table S3 Pearson correlation matrix for samples with pH≥5,6 (n=90)

pH≥5,6 (n=90)	pH	OM	Silt	Sand	Clay	As_{TOT}	Pb_{TOT}	As_G	As_{IN}	Pb_G	Pb_{IN}
**pH**	1										
**OM**	-0,174	1									
**Silt**	-0,051	-0,594[a]	1								
**Sand**	-0,162	0,793**	-0,659**	1							
**Clay**	0,180	-0,267	0,136	-0,680**	1						
**As_{TOT}**	-0,013	-0,686**	0,356	-0,386	0,043	1					
**Pb_{TOT}**	-0,462**	-0,286	-0,109	-0,237	-0,300	0,304*	1				
**As_G**	0,121	-0,596**	0,474	-0,584*	0,019	0,723**	0,437**	1			
**As_{IN}**	-0,082	-0,436	0,799	-0,997**	-0,364	0,443*	-0,072	0,664**	1		
**Pb_G**	-0,400**	-0,195	-0,255	0,097	-0,305	0,070	0,889**	0,416**	-0,132	1	
**Pb_{IN}**	-0,247[a]	-0,659	-0,246	0,346	-0,544*	0,345[a]	0,692**	0,412*	0,415*	0,755**	1

[a]p<0,1
*p<0,05
**p<0,01

As_{TOT}= total concentration of arsenic
Pb_{TOT}= total concentration of lead
As_G= gastric bioaccessibility of arsenic
As_{IN}= intestinal bioaccessibility of arsenic
Pb_G= gastric bioaccessibility of lead
Pb_{IN}= intestinal bioaccessibility of lead

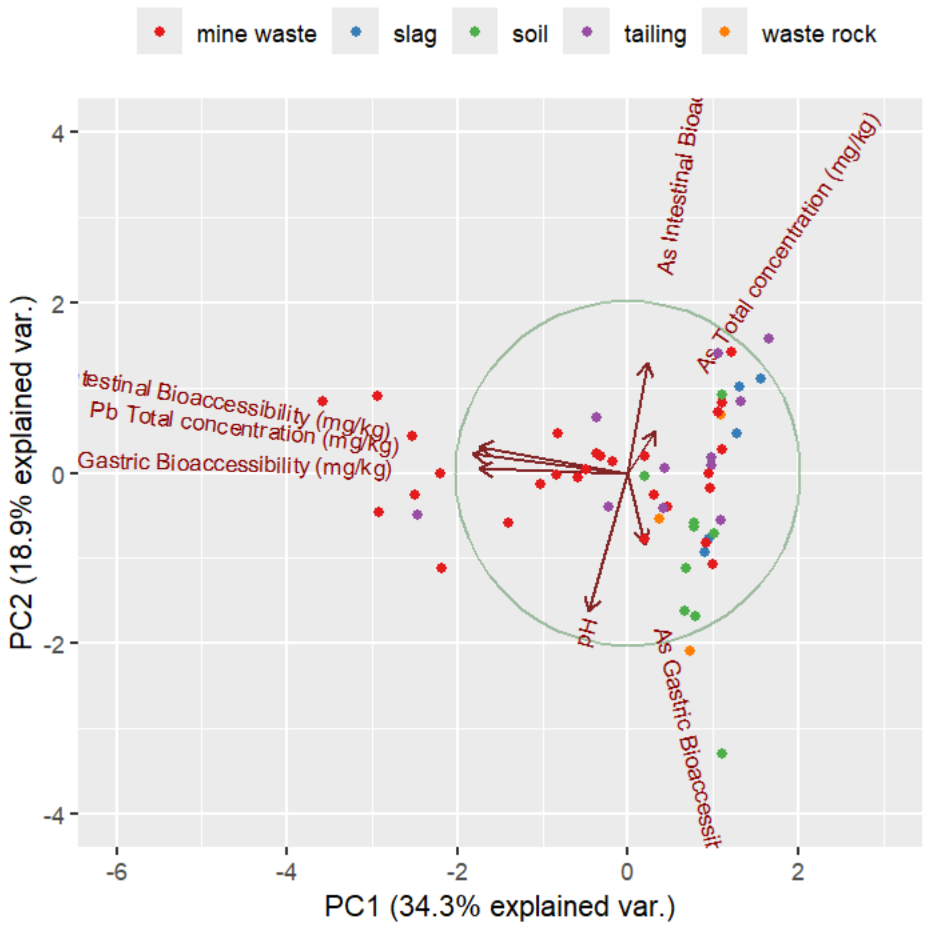

Figure S5 PCA for total As and Pb concentration and bioaccessibility in five types of sample (pH <5.6)

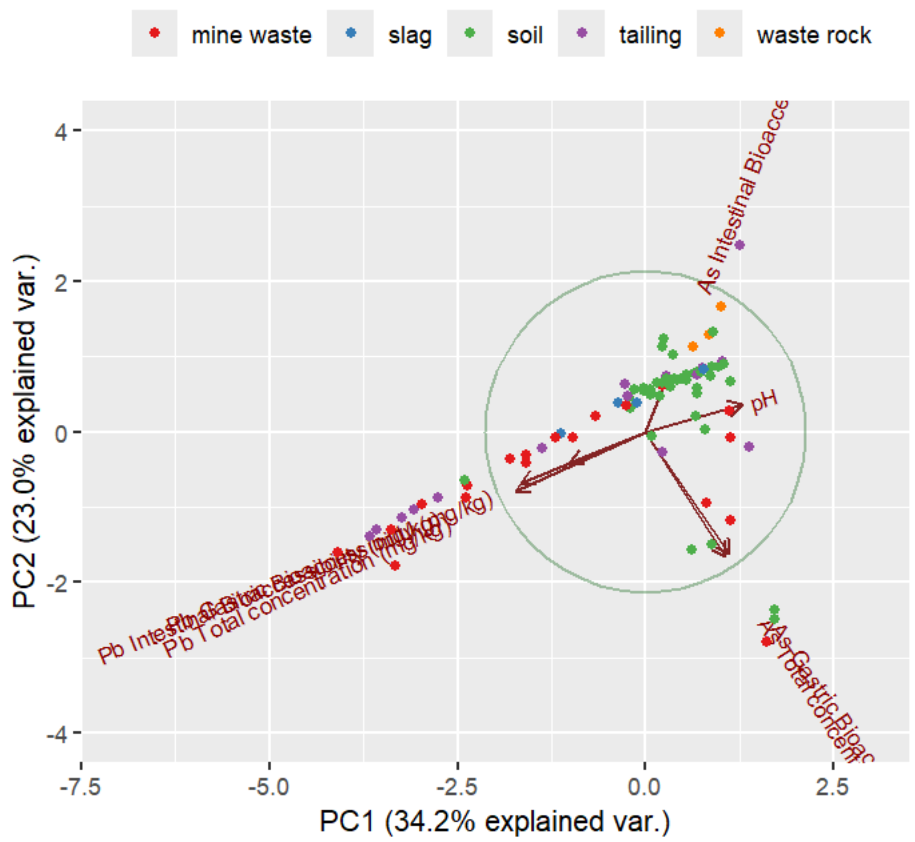

Figure S6 PCA for total As and Pb concentration and bioaccessibility in five types of sample (pH ≥5,6)

Table S4 Stepwise Multiple Linear Regression per sample type, and per 'pH group'

	Sample type	Phase	Regression model	R^2
pH <5,6	**All** (n= 58)	AsG (n=19)	-0,41 + 0,61*AsTOT – 0,93*OM	0,9401**
		AsG (n=35)	0,29 + 0,72*pH	0,1577[a]
		AsIN (n=31)	-2,09 + 0,63*AsTOT + 1,01*AsG	0,5906**
		PbG (n=55)	-3,65 + 4,70*pH + 0,95*PbTOT	0,8291**
		PbIN (n=49)	0,30 – 1,20*PbTOT + 0,71*PbG	0,7239**
	**Mining residue** (n= 49)	AsG (n=15)	-1,20 + 0,30*pH + 0,22*AsTOT + 0,95*Clay	0,6418*
		AsG (n=29)	-0,25 + 0,34*AsTOT	0,1783[a]
		AsIN (n=27)	-1,93 + 0,59*AsTOT + 0,91*AsG	0,5090*
		PbG (n=49)	-3,97 + 5,24*pH + 0,95*PbTOT	0,8221**
		PbIN (n=47)	0,50 – 1,67*pH + 0,21*PbTOT + 0,75*PbG	0,7245**
	**Soil** (n= 9)	AsG (n=6)	-2,02 + 1,01*AsTOT + 0,77*Clay	0,9110**
		AsIN (n=4)	-4,95 + 2,27*AsTOT	0,9792**
		PbG (n=6)	1,76 – 2,42*pH + 0,73*PbTOT	0,9142**
		PbIN (n=2)	///	///
pH ≥5,6	**All** (n= 90)	AsG (n=67)	-3,56 + 3,40*pH + 0,84*AsTOT	0,5449**
		AsIN (n=51)	0,22 + 0,17*pH – 0,40*AsTOT + 1,20*AsG	0,5825**
		PbG (n=68)	-2,17 + 1,80*pH + 1,07*PbTOT	0,7928**
		PbIN (n=48)	0,72 – 0,38*PbTOT + 0,98*PbG	0,6146**
	**Mining residue** (n= 42)	AsG (n=21)	-5,68 + 5,96*pH + 1,04*AsTOT – 0,60*Clay	0,7661**
		AsG (n=28)	-6,56 + 7,11*pH + 0,81*AsTOT	0,4934**
		AsIN (n=22)	-2,93 + 3,97*pH – 0,81*AsTOT + 1,56*AsG	0,8115**
		PbG (n=34)	-6,60 + 5,87*pH + 1,35*PbTOT	0,9**
		PbIN (n=23)	0,52 – 0,27*PbTOT + 0,90*PbG	0,5208**
	**Soil** (n= 48)	AsG (n=24)	0,11 + 0,60*AsTOT – 0,58*OM	0,6991**
		AsG (n=39)	-0,95 + 0,93*AsTOT	0,653**
		AsIN (n=29)	1,93 – 3,67*pH + 0,56*AsTOT	0,5703**
		PbG (n=34)	0,29 – 0,38*pH + 0,84*PbTOT	0,5429**
		PbIN (n=25)	0,88 – 0,65*PbTOT + 1,25*PbG	0,6672**

TOT= total concentration
G= gastric bioaccessibility
IN= intestinal bioaccessibility

*p=0,05
**p=0,001

Table S5 Comparison of six in vitro methods

	specific	**PBET**	**UBM**	**IVG**	**RIVM**	**SBRC**	**RBALP**
		(Ruby, 1996)	(BARGE, 2010) + (Denys, 2012)	(Rodriguez, 1999) + (Schroder, 2004)	(Oomen, 2006)	(Juhasz, 2009)	(Drexler, 2007)
**metal(loid)**		Pb and As	Pb and As	As and Pb	Pb	Pb	Pb
**input**	*amount of material added*	0.4g	0,6g	4g	0-06 or 0,6g	1-10mg/l	1g
	Solid to liquid ratio	1:160	depends on stage	1:150	depends on stage	1:100	1:100
**general**	*type model*				static		
	temperature	37°C	37°C	37°C	37°C	37°C	37°C
	mechanical treatment	argon gas agitation and peristalsis	end-over-end rotation (40rpm)	stirring	end-over-end rotation (55rpm)	suspension mixer (40rpm)	end-over-end rotation (28 rpm)
**oral cavity**	*saliva compartment*	yes	yes	no	yes	no	no
	volume of saliva	40ml	9ml	-	9ml	-	-
	pH	-		-	6,5	-	-
	incubation time	-	5min	-	5 min	-	-
**stomach**	*gastric compartment*	yes	yes	yes	yes	yes	yes
	volume of gastric juice	250ml	13,5ml	600ml	13,5ml		100ml
	pH	2,5	1,2	1,8	1,07	1,5	1,5
	incubation time	1h	1h	1h	2h	1h	1h
	gastric secretion components	0,1% pepsin		1% pepsin			
**intestine**	*intestinal compartment*	yes	yes	yes	yes	yes	no
	volume of intestinal juice	-	duodenal: 27ml bile: 9ml	-	duodenal: 27ml		-
	pH	7	6,3	5,5	duodenal: 7,8	7,5	-
	incubation time	4h	4h	1h	2h	4h	-
	concentration of bile in chyme	70mg bile salts and 20mg pancreatin		2,1g bile and 0,21g pancreatin	9ml (pH8)	1750mg/l bile and 500mg/l pancreatin	
	origin bile	porcine	bovine bile and porcine pepsine and porcine pancreatin	porcine	porcine	bovine bile and porcine pancreatin	
**output**	*centrifugation*	2100g, 25min	4500g, 15min	10000 rpm, 15min, 5211g	3000g, 5min		15ml centrifuge tube
	filtration	dialysis bag-1g NaHCO3 and 2ml DI water		0,45µm filter	no		0,45µm cellulose acetate filter
	destruction		1ml 67% HNO_3		3g soil/6ml DI water + 1ml 65% HNO_3, microwave		
	analytical method	ICP		ICPAES and ICPHG	ICPMS	ICP-AES and ICPMS	ICPAES
